# Reviews and Book Notices

**Published:** 1879-02

**Authors:** 


### Reviews and Book Notices.

ARTICLE XVIII.—CYCLOPÆDIA OF THE PRACTICE OF MEDICINE. Edited by Dr. H. Von Ziemssen, Professor of Clinical Medicine in Munich, Bavaria. Vol. XVII. GENERAL ANOMALIES OF NUTRITION AND POISONS. By Prof. Immermann, of Basel; Prof. R. Boehm, of Dorpat; Prof. B. Naunyn, of Königsberg; and Prof. H. Von Bœck, of Munich. Translated by W. Bathurst Woodman, M. D., and J. Burney Yeo, M. D., of London; Edward S. Wood, M. D., of Boston; Charles Emmerson, M. D., of Concord; Porter Farley, M. D., of Rochester; A. Brayton Ball, M. D., and Elwyn Waller, PH. D., of New York. Albert H. Buck, M. D., of New York, Editor of American Edition. New York: Wm. Wood & Co.

The profession is so familiar with the plan and character of this great work that it has come to anticipate before a new volume is finished what it will be, so that a complete review of the volume before us scarcely seems to be demanded here. We are safe in saying that Vol. XVII. will not fail to meet the highest expectations of the profession. If some of the preceding volumes have been more practical than this, none certainly have been more interesting.

The first part of this volume is devoted to general anomalies of nutrition, by Prof. Immermann. Under this heading are considered in an exhaustive and intensely interesting manner, three diseases—hæmophilia or bleeder's disease, scurvy and purpura hæmorrhagica.

Part second, comprising more than two-thirds of the volume, is devoted to poisons.

Poisoning by metalloids, acids, alkalies, earths and their salts, anæsthetics and other poisonous carbon compounds, and poisoning by tainted articles of diet, is considered by Prof. Boehm.

The article on alcohol poisoning is full and interesting, but alcoholism is not discussed as a distinct disease and no specific is offered for its cure. We were surprised to find that among the remedies for delirium tremens no mention is made of bromide of potassium.

In the discussion of chloroform and ether Prof. Boehm shows a decided preference for the former, as an anæsthetic. He admits that "the published cases of poisoning by ether are very few and bear no comparison at all with the number of cases of poisoning by chloroform. However, this is not so much due to the greater safety of ether, but rather to the fact that while the use of ether has steadily diminished that of chloroform has as constantly increased." We heartily concur in Dr. Edward Curtis' note of criticism where he says, "The enormous clinical experience with ether in America, overwhelmingly disproves the inference made above. There is no escape from the conviction that ether kills so seldom, simply because it is so safe," etc. In the treatment of chloroform or ether poisoning we had thought elevating the feet of the patient of sufficient importance to at least demand notice, but we do not find any mention of it here.

Poisoning by the heavy metals and their salts, including arsenic and phosphorus, is discussed by Prof. Naunyn. Vegetable poisons are considered by Prof. Von Bœck. W. T. M.

ARTICLE XIX. — THE PATHOLOGICAL ANATOMY OF THE EAR. By Herrmann Schwartze, M.D., professor in the University of Halle. With the author's revisions and additions, and with the original illustrations. Translated by J. Orne Green, A.M., M.D., Aural Surgeon, Boston City Hospital, *etc.* Boston: Houghton, Osgood & Co. 1878. Chicago: Jansen, McClurg & Co. 8vo; pp. 174. Price $2.00.

We could not introduce this very interesting book with any better commendation than that of the translator. He says in his preface: "Schwartze's Pathological Anatomy of the Ear" constitutes the sixth part of Kleb's "Handbook of Pathological Anatomy." It is the only comprehensive work strictly devoted to the pathological anatomy of this organ, and on account of the opportunities and devotion of the author in this special field, his well-known thoroughness and strict impartiality in scientific researches, it is a most valuable addition to the literature of otology. It is essentially a hand-book on the subject of which it treats."

The language is concise and lucid ; the text, richly illustrated ; the translation, faultless; and paper and press-work are of that excellent quality so characteristic of the productions of American publishers.

We have read the book with great pleasure, and wish it all the success it justly deserves. F. C. H.

Article XX.—Guy's Hospital Reports. Third series, vol. XXIII. Edited by H. G. Howse, M.S., and Frederick Taylor, M.D.

This volume sustains the reputation of its predecessors. It opens with a paper by Dr. W. A. Brailey, on "Astigmatism in its Relation to Headache and Certain Morbid Conditions of the Eye." He relates fourteen cases showing how the increased effort at accommodation caused various ill effects of such a nature as described in the title, and how they were all relieved by adaptation of proper cylindrical glasses.

Dr. Frederick Taylor, in his illustrated article "On Unilateral Atrophy and Spasm," relates a case of atrophy of one side of the brain, with co-existent atrophy of the other side of the body.

A remarkable feature of the case is that the cerebellum was atrophied on the side opposite to the portion of the cerebrum involved. After some interesting remarks concerning the pathological features of the case, he reports nine cases which had been admitted into the hospital at different times.

Dr. Wilks gives a long account of some unpublished papers left by Dr. Hodgkin. Unfortunately we can not give the detailed summary of many of those which we would like ; suffice it to say that they can only increase the admiration of the reader for the broad attainments and truly scientific character of their writer — pioneer as he was in a great field of pathological research.

Dr. Savage contributes a very readable paper on "The Treatment of Insanity, more especially by Drugs." In it he reviews his experience with the cold shower bath, opium, chloral, camphor chloral, succus conii, hyoscyamia, physostigma, purgatives, stimulants, electricity, etc. He makes some excellent points. Among other notes he thinks he has found physostigma of some slight benefit in cases of general paralysis of the insane.

Mr. Higgens gives a short account of some cases of "Tumor of the Orbit," upon which he had operated.

Mr. Cooper Forster, in his essay on "Stricture of the Urethra," enters a plea for general dilatation, even in most severe cases, claiming that divulsion or section are never necessary, and they are always followed by more or less cicatricial shrinkage.

Dr. Taylor follows with "A Contribution to the History of Idiopathic or Pernicious Anæmia," in which he glances at the early literature of the subject, and then gives a collection of twenty-three cases.

Mr. Jacobson furnishes a description of three cases where he followed the method of supracondyloid amputation of the thigh recommended by Gritti and modified by Stokes. The cases were severe, and he was eminently successful in getting rapid recoveries with very useful stumps. Two plates illustrate his article.

Mr. Golding Bird next follows with three "Selected Cases," one trephining for compression, one of charbon, where there was no bacteria found in the blood, and one of hernia of the ovary—the hernia being inguinal, incomplete and indirect.

Dr. Moxon has a paper on "Chronic Intermittent Albuminuria," in which he describes a mild form of the trouble often met with in adolescents, not accompanied by serious symptoms nor followed by any permanent disorder of functionment.

Dr. Goodhart next contributes some notes on "Some Cases of Diastolic Bruit"; and his article is followed by an essay of over sixty pages, from the pen of Mr. Davy, on "Antiseptic Surgery and Pyæmia." This, as may be surmised, is a plea for the Lister method of dressing; but more than this, the commentary on the five cases reported is excellent, and displays commendable research and reflection. Mr. Lucas contributes notes on the "Termination of a Case of Fracture of the Skull," which he partially reported in Vol. XXI for 1876. This will be read with interest by those who saw his earlier report.

"A Report on Cases of Tetanus treated in Guy's" is the title of the final paper by Dr. Taylor. He reports fifty-one cases treated by one or more of the following: aconite, atropia, bromides (ammonium and potassium), calabar bean, cannabis indica, conium, potassic iodide, nicotine, amyl nitrite and quinia. Of the fifty-one, eight recovered, but it would be difficult to select any one remedy of those mentioned as superior to the rest, judging from the reports, on the whole, though, calabar bean and chloral seem to have given the best results.

Statistical tables, honor lists, etc., close this volume, which no one can read without profit, and which bears out the high reputation that these reports have always borne. R. P.

ARTICLE XXI.—TRANSACTIONS OF THE PHILADELPHIA PATHOLOGICAL SOCIETY. VOL. VII. Edited by J. H. C. Simes. Philadelphia: J. B. Lippincott & Co.

This neat volume of one hundred and seventy pages, comprises about seventy-five articles, being either reports of cases or pathological reports of committees concerning morbid specimens. Interesting points made during the discussion of these cases are also brought out. The volume contains nothing irrelevant—the cases reported are all worthy of record, and the histological reports are concise and models in their way. To notice the articles, even briefly, would be to reproduce much of the volume. Among them, however, we especially noticed "Cases of Injury to the Skull," by Dr. Ashhurst; "Caries of the Sacrum and Trochanter Major," by Dr. H. L. Hodge; "Abdominal Cancer, Obstruction and Double Uterus," by Dr. Starr; "Pancreatic Induration, resembling Scirrhus and Membranous Enteritis with Intestinal Casts," by Dr. Willard; "Embolic Pneumonia," by Dr. Nancrede; "Interstitial Nephritis," by Dr. Hutchinson; "Cases of Extra-uterine Pregnancy," by Drs. Henry and Pepper, with plates illustrating the articles; "Elephantiasis of the Penis," by Dr. Shakespeare; "Anatomy of the Cerebrum," by Dr. Allen; "Cerebral Abscess and Dilated Bronchi," by Dr. Hutchinson; "A Needle found, Post-mortem, within the Cranium," by Dr. Hodge; and the final paper by Dr. Nancrede, on "The Causative Relation Existing between the Anatomical Arrangement of Tissues at Various Ages, and their morbid Growths," a very instructive and readable resumé of the etiology of cancer. R. P.

ARTICLE XXII. — PHYSIOLOGY. PRELIMINARY COURSE LECTURES. By James T. Whittaker, M.A., M.D. Illustrated. Cloth. Pp. 288. Cincinnati: Chancy & Murray. 1879.

This little work was published at the request of many of the author's students, and we do not wonder at the request. As one would infer from the subject, the author does not pretend to give an exhaustive account of the extensive field of physiology, but he does what is equally essential: he inspires his readers with the enthusiasm with which he himself is inspired. He takes up the prominently interesting parts of physiology as a man passionately fond of and well acquainted with children, will take them up and talk to them and about them. The book will be of great interest to the student, and a pleasant recreation to the active practitioner. R. T.

ARTICLE XXIII.—HANDBOOK OF NURSING FOR FAMILY AND GENERAL USE. Published under the direction of the Connecticut Training School for Nurses, State Hospital, New Haven. Philadelphia: J. B. Lippincott & Co. Chicago: Jansen, McClurg & Co. 8vo., pp. 266; price $1.25.

This book was originally intended for use at the school for nurses in the State Hospital at New Haven, Connecticut. But the directors of that school will have no occasion for regret that they allowed the book to be made accessible to everybody. It will do much toward the comfort of the many sick who cannot have the care of a trained nurse; for it teaches, in simple language, every woman how her tenderness must be guided by intelligence and knowledge in rightly performing the duties of a nurse. The rules and instructions laid down in this book are comprehensive and practical. The book gives evidence throughout of being the result of the experience of many years, and will prove a desirable addition to every domestic library.

